# Acute mechanical circulatory support for cardiogenic shock: the “door to support” time

**DOI:** 10.12688/f1000research.11150.1

**Published:** 2017-05-22

**Authors:** Michele L Esposito, Navin K Kapur

**Affiliations:** 1 The Cardiovascular Center, Tufts Medical Center, 800 Washington Street, Boston, Massachusetts, 02339, USA

**Keywords:** ventricular unloading, acute mechanical circulatory support, cardiogenic shock, hemodynamics, percutaneous ventricular assist device

## Abstract

Cardiogenic shock (CS) remains a major cause of in-hospital mortality in the setting of acute myocardial infarction. CS begins as a hemodynamic problem with impaired cardiac output leading to reduced systemic perfusion, increased residual volume within the left and right ventricles, and increased cardiac filling pressures. A critical step towards the development of future algorithms is a clear understanding of the treatment objectives for CS. In this review, we introduce the “door to support” time as an emerging target of therapy to improve outcomes associated with CS, define four key treatment objectives in the management of CS, discuss the importance of early hemodynamic assessment and appropriate selection of acute mechanical circulatory support (AMCS) devices for CS, and introduce a classification scheme that identifies subtypes of CS based on cardiac filling pressures.

## The “door to support” time in cardiogenic shock

Cardiogenic shock (CS) remains a major cause of in-hospital mortality in the setting of acute myocardial infarction (AMI). Several recent reports identified an increase in the prevalence of CS among patients with AMI from 6–7% to 10–12% ^1, 2^. Despite early revascularization, an estimated one in three patients will die during their hospitalization for AMI-CS and one in five patients will die within the first year after discharge for AMI ^3, 4^. More sobering is the fact that over 30% of AMI-CS survivors develop recurrent heart failure (HF) within the first year after discharge ^5^. The natural history of HF is a progressive decline in ventricular function as compensatory remodeling ultimately fails and patients present with recurrent episodes of acutely decompensated HF and ultimately CS owing to advanced HF (CS-HF). A recent analysis of the Interagency for Mechanical Circulatory Support (INTERMACS) registry identified that 52.5% of patients with advanced HF referred for surgical left ventricular (LV) assist device (LVAD) placement present with CS-HF defined as INTERMACS levels 1 or 2 HF ^6^. By 2030, 8 million people in the United States alone will be diagnosed with HF ^7^. Collectively, these data identify CS as a persistent clinical problem and further suggest that the distribution of CS patients may be shifting from CS-AMI to CS-HF over the next decade.

**Figure 1.**
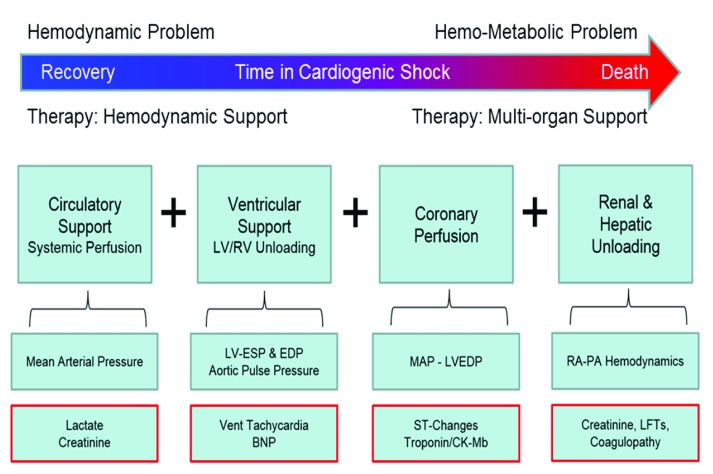
The hemodynamic support equation. The Hemodynamic Support Equation encompasses the four major management objectives for patients with cardiogenic shock, which include: circulatory support, ventricular unloading, myocardial perfusion, and decongestive strategies. BNP, brain natriuretic peptide; CK-MB, creatinine kinase and its MB isozyme; EDP, end-diastolic pressure; ESP, end-systolic pressure; LFT, liver function test; LV, left ventricle; MAP, mean arterial pressure; PA, pulmonary artery; RA, right atrium; RV, right ventricle.

Irrespective of the injurious mechanism, CS begins as a hemodynamic problem with impaired cardiac output leading to reduced systemic perfusion, increased residual volume within both ventricles, and increased cardiac filling pressures. If these hemodynamic derangements persist, reduced tissue perfusion and elevated filling pressures lead to multi-organ ischemia, increased lactate accumulation, hepatic and venous congestion, and worsening multi-organ function ^8^. At this stage, CS has transitioned from a potentially reversible hemodynamic problem to a more complex “hemo-metabolic” problem that may not respond to treatment of the underlying cause or hemodynamic support alone ( Figure 1). For this reason, early identification of CS and application of hemodynamic support in CS may improve clinical outcomes. Rapid triage and treatment algorithms for CS require a similar approach currently employed for ST-segment elevation myocardial infarction (STEMI), whereby early diagnosis, emergent network activation, and short “door to balloon” (DTB) coronary reperfusion times have substantially reduced in-hospital mortality associated with STEMI. For CS, a similar quality metric that reflects the time between onset of CS and initiation of acute mechanical circulatory support (AMCS) should be developed as the “door to support” (DTS) time. Several recent reports support the concept of a DTS time and have observed improved survival with early initiation of AMCS before percutaneous coronary revascularization or before the initiation of inotropes and vasopressors in the setting of AMI-CS ^9– 11^. Future studies quantifying the optimal DTS time in CS are required.

## The hemodynamic support equation

A critical step towards the development of future algorithms is a clear understanding of the treatment objectives for CS. These four primary objectives are summarized in the “hemodynamic support equation” and include 1) circulatory support, 2) ventricular unloading, 3) myocardial perfusion, and 4) decongestion ( Figure 1). Adequate circulatory support is defined by an increase in mean arterial pressure and enhanced microvascular organ perfusion. Ventricular unloading is defined as a reduction in myocardial work and wall stress, which is best achieved by reducing native ventricular pressure and volume ^12^. Myocardial perfusion is defined as increased epicardial and microvascular coronary blood flow and is often associated with successful circulatory and ventricular support. Decongestion refers to a reduction in total body volume and elevated venous filling pressures, which is commonly associated with worsening renal function, hepatic failure, bowel edema, and subsequent sepsis. To solve the hemodynamic support equation, all four objectives must be achieved in a timely manner.

Pharmacologic approaches fail to solve the hemodynamic support equation. Often drug therapy will solve one part of the equation but at the cost of another. For example, early use of vasopressors such as norepinephrine in CS may increase mean arterial pressure but not microvascular organ perfusion. Furthermore, increased mean arterial pressure will increase LV afterload, thereby increasing myocardial work and wall stress, which promotes myocardial ischemia, impairs cardiac function, and increases cardiac filling pressures. Similarly, inotropic therapy in CS may increase mean arterial pressure but directly increases myocardial work, thereby potentially worsening myocardial ischemia. For these reasons, CS refractory to one or more vasopressors or inotropes is associated with increased in-hospital mortality.

## Solving the hemodynamic support equation with acute mechanical circulatory support devices

**Figure 2.**
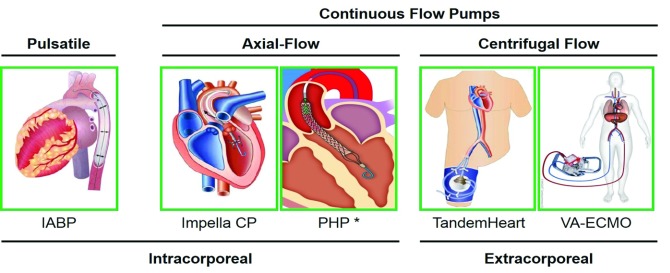
Left ventricular acute mechanical circulatory support devices. Contemporary acute mechanical circulatory support devices for left ventricular support are illustrated and categorized by mode of action (pulsatile or continuous-flow pumps), type of rotary flow pump (axial- or centrifugal flow), and pump location (intracorporeal or extracorporeal). IABP, intra-aortic balloon counter-pulsation pump; PHP, percutaneous heart pump; VA-ECMO, veno-arterial extracorporeal membrane oxygenation.

In the contemporary era, the hemodynamic support equation can be readily addressed with early and appropriate use of AMCS devices, which can be broadly categorized by their mechanism of action as pulsatile or rotary flow pumps ( Figure 2).

The intra-aortic balloon counter-pulsation pump (IABP) is a catheter-mounted balloon that augments pulsatile blood flow by inflating during diastole, thereby increasing diastolic pressure in the aortic root and enhancing coronary blood flow, while also displacing blood volume in the descending aorta. During systole, rapid deflation of the intra-aortic balloon generates a pressure sink, which reduces LV afterload and increases LV cardiac output ^13^. The magnitude of hemodynamic support generated by an IABP is directly related to LV cardiac output. Recent studies confirm that the more dysfunctional the LV, the less effective an IABP becomes ^14– 16^. In 2012, the IABP-SHOCK II study reported no benefit with IABP therapy in patients with AMI-CS. No large, randomized studies have evaluated the utility of IABP therapy in HF-CS ^17^.

In contrast to counter-pulsation balloons, rotary-flow pumps generate rotational kinetic energy, which increases blood flow. Rotary flow pumps can be further categorized based on the type of motor as axial-flow or centrifugal-flow systems ^18^. Axial-flow AMCS pumps are placed across the aortic valve and displace blood from the LV into the ascending aorta. The net result of these trans-valvular axial pumps is a reduction in LV pressure and volume with a concomitant increase in mean aortic root pressure. As a result, systemic perfusion is increased, LV wall stress is reduced, and the trans-myocardial perfusion gradient (aortic diastolic pressure – LV diastolic pressure) is increased. Furthermore, several prior studies have shown that under ischemic conditions, coronary blood flow is increased after activation of a trans-valvular axial-flow pump ^19, 20^. Trans-valvular axial-flow pumps directly solve three of the four major objectives in the hemodynamic support equation by increasing mean arterial pressure, reducing LV pressure and volume, and increasing coronary blood flow. Contemporary trans-valvular axial-flow pumps include the Impella (Abiomed Inc, Danvers, MA) or the HeartMate percutaneous heart pump (PHP) (Abbott Inc, Chicago IL) ^21, 22^. The PHP device is currently under investigation in the United States as part of the SHIELD II trial. The Impella devices are the only AMCS pumps approved by the US Food and Drug Administration for use in CS.

Centrifugal-flow pumps include the TandemHeart device (TandemLife, Pittsburgh, PA) and veno-arterial extracorporeal membrane oxygenation (VA-ECMO) ^22^. The TandemHeart and VA-ECMO systems draw blood from the left or right atrium, respectively, into an extracorporeal pump that displaces the blood into the femoral artery, thereby pressurizing the arterial tree and increasing mean arterial pressure. Since VA-ECMO displaces venous blood into the arterial system, an oxygenator is placed in the circuit prior to the return of blood to the femoral artery. The distinct location of the inflow cannula has a profound impact on the hemodynamic effects of these two systems ^23^. Since VA-ECMO drains blood from a large venous reservoir, at typical flow rates of 4 to 6 liters/minute, VA-ECMO does not significantly reduce LV volume. As a result, VA-ECMO increases LV pressure, wall stress, and myocardial work and fails to solve the hemodynamic support equation. In contrast to VA-ECMO, by displacing blood from the left atrium, the TandemHeart device effectively reduces LV preload, thereby reducing LV volume, wall stress, and workload, while increasing systemic mean arterial pressure and myocardial perfusion ^24^. The TandemHeart system is able to solve the same three objectives of the hemodynamic support equation as do trans-valvular axial-flow pumps; however, a major technical limitation of the TandemHeart device is the need for a puncture across the interatrial septum to deliver the 21 French cannula that drains the left atrium.

In summary, the trans-valvular axial-flow pumps and the TandemHeart left atrial-to-femoral artery centrifugal-flow pump successfully achieve three of the four major objectives of the hemodynamic support equation: circulatory support, ventricular unloading, and enhanced coronary perfusion.

## Right ventricular acute mechanical circulatory support devices

**Figure 3.**
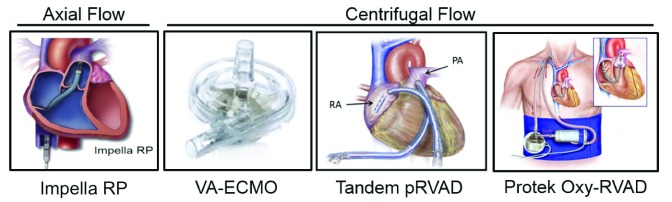
Right ventricular acute mechanical circulatory support devices. Contemporary acute mechanical circulatory support devices for right ventricular support are illustrated and categorized by type of rotary flow pump (axial- or centrifugal-flow). pRVAD, percutaneous right ventricular assist device; VA-ECMO, veno-arterial extracorporeal membrane oxygenation.

Over the past 5 years, the introduction of right ventricular (RV) non-surgical AMCS devices has advanced our ability to support patients with CS and either isolated RV failure or biventricular (BiV) failure. Options for RV-AMCS include the Impella RP, the TandemHeart RVAD, and VA-ECMO ( Figure 3). The Impella RP and TandemHeart RVAD function by displacing blood from the right atrium (RA) to the pulmonary artery, whereas VA-ECMO drains the RA and displaces blood into the arterial system. The use of RV-AMCS devices has increased awareness of RV dysfunction in the setting of AMI, CS, and HF and after LVAD surgery. While clinical reports support the hemodynamic effects of RV-AMCS ^25– 27^, no guidelines regarding their use have been developed to date.

## Decongestion in cardiogenic shock: an important target of therapy

A critical barrier to successful clinical outcomes in advanced HF and CS is persistent systemic volume overload or congestion. Recent studies have identified that elevated right heart filling pressures are directly related to worsening renal function and further that elevated BiV filling pressures are associated with increased short-term mortality ^28, 29^. In CS, adequate circulating volume is necessary to maintain cardiac output; however, excess circulating volume may be detrimental to multi-organ function. As described above, AMCS devices can effectively address parts of the hemodynamic support equation. However, in isolation, AMCS devices alone cannot address the fourth objective, namely, decongestion. Decongestive approaches such as concomitant diuretic therapy or renal replacement therapy should be considered early in CS for patients with elevated BiV filling pressures refractory to diuretics and AMCS device support.

## Hemodynamic profiles in cardiogenic shock

The contemporary definition of CS must evolve beyond metrics associated with the early stages of hemo-metabolic shock such as hypotension and evidence of low perfusion, including cold and clammy extremities and end-organ dysfunction ^19, 30^. At this stage, CS is becoming irreversible. Emerging evidence supports the use of pulmonary artery catheters (PACs) to identify CS before metabolic failure ensues and to define the hemodynamic condition of patients in advanced HF and CS ^31^. PAC guidance must be strongly considered in patients with suspected CS to confirm the presence of CS (low cardiac output), define the congestive profile in CS (cardiac filing pressures), and to evaluate the patient’s response to therapeutic interventions.

**Figure 4.**
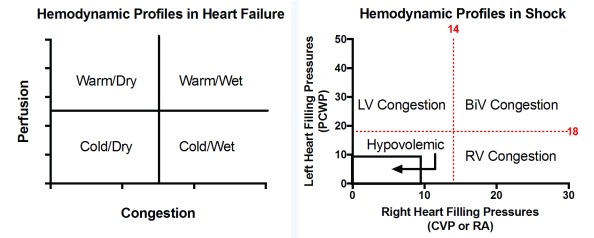
Congestive profiles in cardiogenic shock. Clinical assessment of hemodynamic conditions in decompensated heart failure is traditionally categorized into four groups based on systemic perfusion and congestive status using a two-by-two table. We now propose a similar two-by-two construct to define hemodynamic profiles in cardiogenic shock based on congestive state using measures of left and right heart filling pressures. Cardiogenic shock is categorized as having LV-, RV-, or BiV-dominant congestion or hypovolemia. Treatment approaches may be tailored to each of these four categories. BiV, biventricular; CVP, central venous pressure; LV, left ventricular; PCWP, pulmonary capillary wedge pressure; RA, right atrial; RV, right ventricular.

Early acquisition of hemodynamic data also helps to define CS as univentricular or BiV. Beginning in the early 1980s, several studies identified the importance of right and left heart filling pressures in AMI, CS, and advanced HF ^32– 34^. The relationship between RA and pulmonary capillary wedge pressure (RA:PCWP ratio) has been used to identify RV failure in AMI and is associated with prognosis in advanced HF. Analogous to the 2×2 evaluation of patients with advanced HF as being “warm or cold and dry or wet” ^35^, the RA:PCWP ratio allows us to classify CS based on congestive state into four hemodynamic profiles: hypovolemic, LV-, RV-, or BiV-dominant congestion ^36^. For patients with CS failing to improve despite the initiation of one vasopressor or inotrope, each of these four categories may require a different therapeutic approach ( Figure 4). The hypovolemic-CS patient may require volume resuscitation. The LV-CS or RV-CS patients may require specific approaches to modulate univentricular preload or afterload or treatment with a left- or right-sided AMCS device, respectively. The BiV-CS patient may require more aggressive decongestive therapy along with LV or BiV AMCS therapy. Future studies are required to determine whether defining CS based on hemodynamic profile alters management strategies and leads to improved clinical outcomes. Now is the time for a series of prospective, randomized trials or prospective registries confirming the clinical utility of hemodynamic assessment and AMCS device therapy in CS. One recently launched prospective registry is the Detroit Shock Initiative, which involves early application of the Impella trans-valvular axial-flow pump in the setting of AMI-CS ^37^.

In conclusion, as our options to stabilize and rescue patients from the slippery slope of hemodynamic to hemo-metabolic CS grow, we must develop new guidelines that involve 1) early hemodynamic assessment of CS, 2) early use of AMCS devices for refractory CS, 3) identification of the optimal DTS time, 4) appropriate AMCS device selection based on the clinical scenario, and 5) early use of decongestive therapies to reduce the propensity for worsening metabolic failure despite adequate circulatory support.
